# A patient perspective on enduring symptoms – the unmet need

**DOI:** 10.1016/j.fhj.2025.100465

**Published:** 2025-12-15

**Authors:** Katharine Cheston

**Affiliations:** Mildred Blaxter Postdoctoral Fellow, Department of Sociology & Institute for Medical Humanities, University of Durham, UK

## Abstract

This short paper illustrates the lived experience of individuals with severe enduring symptoms: chronic, often debilitating conditions for which no clear medical explanation currently exists. Drawing on qualitative interviews, the paper highlights the profound suffering, isolation, and lack of medical support experienced by this underserved population. It examines the systemic barriers to care, including stigma, the absence of follow-up services, and the traumatising nature of some healthcare encounters, which can lead to healthcare avoidance even in the face of potentially life-threatening symptoms. It concludes with a call for improved training for clinicians, increased capacity within NHS services, and ring-fenced funding for biomedical research.

## The scale of the suffering

Since 2019, my research has focused on ‘enduring symptoms’. These are chronic, often debilitating symptoms and syndromes for which medical evidence of disease cannot yet be found; they are typically described in the clinical literature as ‘medically unexplained’ or ‘functional’ symptoms.

In this short paper, I will illustrate the patient perspective by drawing on interviews I conducted with those who live with severe enduring symptoms and who are either housebound or bedbound. While these qualitative data are not representative of all those who live with these illnesses – many of whom will experience less severe, and hopefully less enduring, symptom presentations – this perspective offers an invaluable insight into the unmet needs of this patient population.

When I began my research, I was well aware that enduring symptoms can (as the authors of one review paper recently stated) ‘cause far greater disability than diseases where signs are prominent’.^1^ Encountering the reality of this suffering first-hand was still shocking and deeply saddening. One of my interviewees, who had lived with enduring symptoms for over 3 decades, had all her teeth removed as the severity of her illness meant that she had been unable to care for them. Multiple interviewees spoke of their distress at only being able to take a shower once every couple of months.

The scale of the suffering also had practical consequences for the research process: I had to devise a method of interviewing that was accessible for those who were severely unwell. For example, one of my interviewees – a woman in her twenties living with a range of enduring symptoms – completed her interview by sending me short voice notes; collecting 90 min of interview testimony was a process that took 18 months, due to the significant limitations her illness placed on her.

## Lack of care and support

Compounding this suffering is the lack of support. With no medical explanation for their symptoms, many struggle to receive appropriate care. One interviewee told me that all she’d eaten the previous day was a bowl of cereal; without family help or a social care package, this was all she had been able to prepare for herself. Stigma, too, is a significant barrier to care. Far from rallying around and providing support and sympathy, family and friends can be unsympathetic or even unkind. NHS healthcare, too, often seems to stop when tests come back ‘normal’. ‘There’s just no follow-up care’ was a regular refrain I heard while carrying out my interviews. With no regular appointments or reviews, those with enduring symptoms can be left managing severe symptoms – which limit their calorie intake and capacity for personal care – alone, and often completely unsupported.

The minutes of a recent All-Party Parliamentary Group meeting highlighted the stark reality of this situation, when a respiratory consultant detailed a case of an 18-year-old who has been bedbound for nearly 3 years due to long COVID and myalgic encephalomyelitis / chronic fatigue syndrome (ME/CFS).^2^ Fed through a tube and unable to sit up for more than a few minutes without losing consciousness, they have no regular NHS support apart from occasional contact with a general practitioner (GP) and dietician. As one of my interviewees, also living with ME/CFS, put it:

‘to have your life absolutely turned upside down and kind of be left, really… it’s just really awful and could be so much better, and should be so much better’.

## Avoidance of healthcare

People with enduring symptoms are often thought of as ‘frequent attenders’ of health services – and studies show that high users of healthcare are more likely to experience symptoms that are unexplained by disease, when compared with low-use groups.^3^, ^4^, ^5^, ^6^ But there is a gaping hole in the literature: there are no large studies examining health service attendance across the population of people who experience enduring symptoms, and my qualitative data suggest that a proportion of these patients have actually disengaged from NHS services.

I found that those living with severe enduring symptoms are more likely to avoid consulting healthcare professionals. For some, this avoidance stemmed from a sense of futility. They felt that it was pointless contacting their GP if new symptoms emerged or existing symptoms worsened, presuming that they would be unable to help: ‘You just give up and treat yourself’, one interviewee told me. Others avoided healthcare because their previous experiences had been so distressing and even (as one interviewee put it) ‘traumatising’. In these cases, self-managing their symptoms – even when they know that these symptoms are concerning – can feel safer than engaging with NHS services. One interviewee, following an upsetting interaction with a consultant, opted to forgo medical care – even when she developed new symptoms that she knew could be red flags for a potentially life-threatening condition. For another, the prospect of having to consult a GP with symptoms of a urinary tract infection (UTI) was so frightening that she delayed seeking help until she collapsed and required hospital treatment.

Healthcare avoidance in this patient population is far from uncommon, and while it is now starting to be acknowledged in the academic literature,^7^ this phenomenon is well recognised within patient communities: a 2019 survey by Action for ME, for example, revealed that 64% of respondents did not currently see any NHS healthcare professionals about their ME/CFS.^8^

## Conclusion: creating change

The sheer magnitude of the unmet needs is devastating: this population live with disabling and life-limiting symptoms, yet they feel alone and abandoned, receiving little to no institutional support, care or recognition of their suffering. I know all too well how difficult it is to witness the scale of the suffering that this patient group endures – and how easy it is to feel powerless. But there are changes that could be made that would improve the lives of those living with these enduring symptoms. Developing a training programme for clinicians could prevent the kind of traumatising clinical encounters that left some of my interviewees feeling they needed to avoid healthcare at all costs. Increasing capacity within the NHS to provide consultant oversight for those living with severe enduring symptoms could be transformative in ensuring that these vulnerable patients no longer fall through cracks in services identified, for example, in the Prevention of Future Deaths report produced after the inquest into the death of Maeve Boothby O’Neill.^9^ Finally, the impressive initial results of the DecodeME study,^10^ which revealed a potential genetic predisposition to developing ME/CFS, indicate the huge potential for discovery in this field; providing dedicated, ring-fenced funding for biomedical research could produce the answers that this patient group desperately need and deserve.

## CRediT authorship contribution statement

**Katharine Cheston:** Writing – original draft.

## Declaration of competing interest

The authors declare the following financial interests/personal relationships which may be considered as potential competing interests: Katharine Cheston reports financial support was provided by the Wellcome Trust and by the Foundation for the Sociology of Health and Illness. If there are other authors, they declare that they have no known competing financial interests or personal relationships that could have appeared to influence the work reported in this paper.
